# Combined vascular endothelial growth factor-A and fibroblast growth factor 4 gene transfer improves wound healing in diabetic mice

**DOI:** 10.1186/1479-0556-8-6

**Published:** 2010-08-30

**Authors:** Agnieszka Jazwa, Paulina Kucharzewska, Justyna Leja, Anna Zagorska, Aleksandra Sierpniowska, Jacek Stepniewski, Magdalena Kozakowska, Hevidar Taha, Takahiro Ochiya, Rafal Derlacz, Elisa Vahakangas, Seppo Yla-Herttuala, Alicja Jozkowicz, Jozef Dulak

**Affiliations:** 1 Department of Medical Biotechnology, Faculty of Biochemistry, Biophysics and Biotechnology, Jagiellonian University, Krakow, Poland; 2 Section for Studies on Metastasis, National Cancer Center Research Institute, Tokyo, Japan; 3 Research & Development Department, Adamed Ltd., Pienkow, Poland; 4 A. I. Virtanen Institute, University of Kuopio and Gene Therapy Unit, Kuopio University Hospital, Kuopio, Finland

## Abstract

**Background:**

Impaired wound healing in diabetes is related to decreased production of growth factors. Hence, gene therapy is considered as promising treatment modality. So far, efforts concentrated on single gene therapy with particular emphasis on vascular endothelial growth factor-A (VEGF-A). However, as multiple proteins are involved in this process it is rational to test new approaches. Therefore, the aim of this study was to investigate whether single AAV vector-mediated simultaneous transfer of VEGF-A and fibroblast growth factor 4 (FGF4) coding sequences will improve the wound healing over the effect of VEGF-A in diabetic (db/db) mice.

**Methods:**

Leptin receptor-deficient db/db mice were randomized to receive intradermal injections of PBS or AAVs carrying β-galactosidase gene (AAV-LacZ), VEGF-A (AAV-VEGF-A), FGF-4 (AAV-FGF4-IRES-GFP) or both therapeutic genes (AAV-FGF4-IRES-VEGF-A). Wound healing kinetics was analyzed until day 21 when all animals were sacrificed for biochemical and histological examination.

**Results:**

Complete wound closure in animals treated with AAV-VEGF-A was achieved earlier (day 19) than in control mice or animals injected with AAV harboring FGF4 (both on day 21). However, the fastest healing was observed in mice injected with bicistronic AAV-FGF4-IRES-VEGF-A vector (day 17). This was paralleled by significantly increased granulation tissue formation, vascularity and dermal matrix deposition. Mechanistically, as shown *in vitro*, FGF4 stimulated matrix metalloproteinase-9 (MMP-9) and VEGF receptor-1 expression in mouse dermal fibroblasts and when delivered in combination with VEGF-A, enhanced their migration.

**Conclusion:**

Combined gene transfer of VEGF-A and FGF4 can improve reparative processes in the wounded skin of diabetic mice better than single agent treatment.

## Introduction

Optimum healing of a cutaneous wound requires a well orchestrated integration of the complex biological and molecular events of cell migration and proliferation, extracellular matrix (ECM) deposition, angiogenesis and remodeling [1,2]. One of the most common disease states associated with impaired tissue repair is diabetes mellitus [1]. Many factors contribute to chronic, non-healing diabetic wounds, among which crucial is the impairment in the production of cytokines and growth factors, such as keratinocyte growth factor (KGF), vascular endothelial growth factor-A (VEGF-A) or platelet-derived growth factor (PDGF) by local inflammatory cells and fibroblasts [1,3,4].

In animal models of impaired wound healing diminished neovascularization is also associated with delayed or diminished production of VEGF-A and other angiogenic growth factors [5]. VEGF-A, as the most potent angiogenic factor of the VEGF family members, exerts its mitogenic activity via its receptors VEGF-R1 (Flt-1) and VEGF-R2 (Flk-1), which are expressed mainly by endothelial cells [6]. Moreover, VEGF-A may modulate expression of plasminogen activator (PA) and plasminogen activator inhibitor-1 (PAI-1) in microvascular endothelial cells [7] as well as influence endothelial cell-derived matrix metalloproteinases (MMPs) activity [8]. These actions contribute to the ability of VEGF-A to promote endothelial cell invasion. Accordingly, it has been shown that VEGF-A delivered either as a protein [9] or as a gene [10,11] improves wound healing in diabetic mice through the stimulation of angiogenesis, re-epithelialization, synthesis and maturation of extracellular matrix.

Fibroblast growth factors (FGFs), a large family of more than 20 multifunctional proteins, stimulate proliferation in a wide range of cell types, through their binding to cell membrane tyrosine kinase receptors [12]. These FGF receptors (FGFRs) comprise 4 receptor tyrosine kinases designated FGFR-1, FGFR-2, FGFR-3, and FGFR-4 [13]. Upon receptor binding, FGFs can elicit a variety of biological responses, such as cell proliferation, differentiation and migration. These activities are critical to a wide variety of physiological as well as pathological processes including angiogenesis, vasculogenesis, wound healing, tumorigenesis, and embryonic development [14].

FGF4 is a member of FGFs family and was the first one among all FGFs to be described as an oncogene. It is expressed during early limb development and throughout embryogenesis [15,16]. In adults, FGF4 is found primarily in tumors, such as stomach cancer, Kaposi sarcoma, and breast cancer [17], but also to some extend in the nervous system, intestines, and testes [18]. Few years ago, also the potential therapeutic application of this growth factor has been highlighted as it has been demonstrated to play a pivotal role in the growth of newly formed capillaries and their enlargement in the process called arteriogenesis [19]. The angiogenic effects of FGF4 are related to the up-regulation of the endogenous VEGF-A expression [19,20]. Unlike FGF-1, -2, and -9, which lack a signal peptide (but may still be released by an alternative secretion pathway), FGF4 is efficiently secreted [21], what is rather advantageous over the other FGFs for the gene therapy. FGF4 protein is a potent mitogen for a variety of cell types of mesodermal and neuroectodermal origin, including fibroblasts and melanocytes [14]. It has also been shown to stimulate endothelial cell proliferation, migration, and protease production *in vitro *and neovascularization *in vivo *[22]. FGFR-2 is the preferred receptor for FGF4 under restricted heparan sulfate conditions [23]. Furthermore, FGF4 similarly to VEGF-A [6], binds to heparan sulfate of the extracellular matrix, what leads to its deposition near the place of synthesis [23].

So far, all efforts concentrated on single gene therapy for the treatment of impaired wound healing. However, as multiple proteins are involved in this process there might be a need to efficiently deliver more than one gene. The role of VEGF-A in the promotion of wound closure has been well documented whereas the effect of FGF4 has not been analyzed. Therefore, the aim of this study was to investigate whether FGF4 will accelerate the wound closure and whether combined AAV-mediated gene therapy approach with VEGF-A and FGF4 coding sequences will improve the wound healing over the effect of VEGF-A in genetically diabetic mice.

## Materials and methods

### Reagents

Cell culture reagents, Dulbecco's Modified Eagle's Medium (DMEM) and foetal bovine serum (FBS) were from PAA (Lodz, Poland). Recombinant human vascular endothelial growth factor (rhVEGF-A) and recombinant human fibroblast growth factor (rhFGF4) as well as hVEGF-A- and hFGF4-recognizing ELISA kits were procured from R&D Systems Europe (Warszawa, Poland). Oligo(dT) primers, dNTPs, MMLV reverse transcriptase, β-galactosidase Enzyme Assay System and Bromodeoxyuridine (BrdU) incorporation assay were obtained from Promega (Gdansk, Poland). pAAV-MCS and pAAV-LacZ plasmid vectors were obtained from Stratagene (Piaseczno, Poland). Proliferating cell nuclear antigen (PCNA) recognizing primary antibodies (clone PC10) and Animal Research Kit (ARK) Peroxidase were procured from DAKO (Gdynia, Poland). Streptavidin Alexa Fluor 546 and Alexa Fluor 488 secondary antibodies were obtained from Invitrogen (Warszawa, Poland). All other reagents and chemicals, unless otherwise stated, were purchased from Sigma (Poznan, Poland).

### AAV vector preparation and characterization

**Figure 1:**
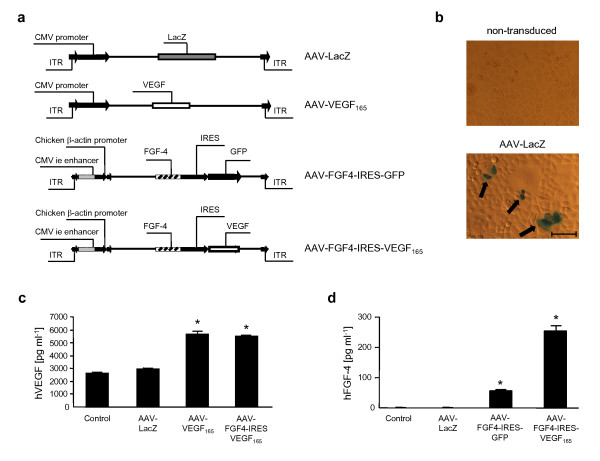
***In vitro *gene expression in AAV-transduced HeLa cells**. (A) Schematic representation of expression cassettes in AAV vectors used for transduction: control vector encoding β-galactosidase - AAV-LacZ; VEGF-A overexpressing vector - AAV-VEGF-A; FGF4 (cap-dependent cistron) and GFP (IRES-dependent cistron) - AAV-FGF4-IRES-GFP; FGF4 (cap-dependent cistron) and VEGF-A (IRES-dependent cistron) - AAV-FGF4-IRES-VEGF-A. CMV ie enhancer - cytomegalovirus immediate-early enhancer. IRES - internal ribosome entry site. (B) β-galactosidase *in situ *staining of non-transduced or AAV-LacZ-transduced HeLa cells (arrows). (C) and (D) ELISA determining respectively, hVEGF-A and hFGF4 release into the cell culture media. Production of both hVEGF-A and hFGF4 proteins was significantly up-regulated after transduction with therapeutic vectors when compared to non-transduced (control) cells or cells transduced with AAV-LacZ vector. Representative data out of two independent experiments performed in duplicates. Values are means ± SD; *p < 0.05 vs control and AAV-LacZ. Scale bar = 0.1 mm.

Four AAV serotype 2 vectors (AAV2) were used in the present study (Figure 1a). They were carrying either LacZ reporter (control) gene under the control of constitutive CMV (cytomegalovirus) immediate early promoter or human 165-isoform of VEGF-A under the control of strong CMV promoter or human FGF4 under the control of chicken β-actin promoter and CMV enhancer. Bicistronic vector was carrying human FGF4 and human VEGF-A genes separated by internal ribosomal entry side (IRES) region under the control of chicken β-actin promoter and CMV enhancer. IRES of the Polyoma virus 1 origin permitted simultaneous overexpression of both genes. The cDNA for human VEGF-A was obtained from pSG5-VEGF-A [24] cloned into the pAAV-MCS. pTR-UF12 and pTR-UF22 were used for cloning of bicistronic plasmid vectors carrying FGF4 and GFP or FGF4 and VEGF-A respectively, and were kindly gifted by Dr Sergei Zolotukhin [25]. cDNA for human FGF4 was subcloned by PCR with appropriate primer pairs from pCAGGS-HST plasmid [26].

Infectious vector stocks were generated in HEK-293 cells (human embryonic kidney-293 cells), cultured in 150-mm diameter Petri dishes, by co-transfecting each plate with 15 μg of each vector plasmid, together with 45 μg of the packaging/helper plasmid pDG (kindly provided by Dr Jurgen A. Kleinschmidt, Program of Infection and Cancer, German Cancer Research Center; Heidelberg, Germany) expressing AAV and adenovirus helper functions. At 12 h after transfection, the medium was replaced with fresh medium and 3 days later the cells were harvested by scraping, centrifuged and the cell pellets resuspended in 15 ml of 150 mM NaCl, 50 mM Tris-HCl (pH 8.5). Three rounds of fast freeze-thawing were performed on the cell lysate and 50 U ml^-1 ^benzonase was added and incubated for 1 h at 37°C. The lysate was then centrifuged at 5 000 rpm for 20 min and supernatant retained and transferred to an Optiseal ultracentrifuge tube (Beckman). An iodixanol gradient was established with 15, 25, 40 and 57% iodixanol (Optiprep); the 25 and 57% fractions contained phenol red so that the 40% fraction, which contained the AAV, was easily visualized. Ultracentrifugation of the gradient was performed in a Beckman ultracentrifuge (rotor type Ti50.2) at 40 000 rpm for 2 h 40 min at 18°C. The 40% fraction (about 3 ml) was removed using a 21G needle and applied to a 1 ml Heparin HP column (Amersham Biosciences) connected to the high-performance liquid chromatography (HPLC) system. The column was washed in 1×PBS-MK (1×PBS, 1 mM MgCl_2_, 2.5 mM KCl) and virus was eluted in 0-1 M gradient of Na_2_SO_4 _in 1×PBS-MK. The viral preparation was desalted by dialysis (Slyde-A-Lyser, Pierce) against 1×PBS at 4°C and stored at -80°C. AAV titer was determined by measuring the copy number of the viral genomes in dialyzed samples. This was achieved by a real-time PCR procedure using primers mapping in the target gene coding region. Primers recognizing LacZ (5'-AGA-ATCCGACGGGTTGTTACTCGC-3' and 5'-TGCGCTCAGGTCAAATTCAGACGGC-3'), hVEGF-A (5'-ATGTCTATCAGCGCAGCTACTGCC-3' and 5'-AGCTCATCTCTCCTAT-GTGCTGGC-3') and hFGF4 (5'-TGGTGGCGCTCTCGTTGGCG-3' and 5'-ATCGGTGA-AGAAGGGCGAGCC-3') were used. The purified viral preparations used in the present study had particle titers of approx. 1 × 10^11 ^viral particles (vp) ml^-1^. Cells in culture and animals received the dose of AAV stated in the experimental protocol.

### Cell culture

HeLa cells (human epithelial cells from a fatal cervical carcinoma) were maintained in low glucose (5.5 mM) DMEM supplemented with 10% heat-inactivated FBS, L-glutamine (2 mM), penicillin (100 U ml^-1^) and streptomycin (10 μg ml^-1^).

Primary isolates of dermal fibroblasts were harvested from 10-week-old diabetic (db/db) C57BLKS mice and their wild-type (WT) littermates. The animals were sacrificed and trunk skin was removed by sharp dissection under sterile conditions. The harvested skin was then minced and digested for 3 hours (from db/db mice) and for 6 hours (from WT mice) in 0.2% collagenase type II (Gibco; Warszawa, Poland) solution in serum-free low glucose DMEM at 37°C. The dissociated cells were then centrifuged and resuspended in low glucose (5.5 mM) DMEM medium supplemented with 20% FBS, 2 mM L-glutamine, 100 U ml^-1 ^penicillin, and 10 μg ml^-1 ^streptomycin. The cells were cultured at standard conditions: 5% CO_2_, 37°C and humidified atmosphere. After the first or second passage cells from diabetic animals were grown either in low (5.5 mM) or in high (25 mM) glucose concentration for 48-72 hours. Fibroblasts from WT mice were cultured in low glucose DMEM. Cells at passage 2 or 3 were used for experiments.

### AAV-mediated transduction of cells in culture

HeLa cells were cultured at density 1 × 10^3 ^per 1 well of the 96-well plate and exposed to 1 × 10^3 ^MOI (multiplicity of infection) of AAV-LacZ, AAV-FGF4-IRES-GFP, AAV-VEGF-A and AAV-FGF4-IRES-VEGF-A for 72 hours. After that time the transduction efficiency was determined by β-galactosidase *in situ *staining and conditioned culture media were collected for the measurement of therapeutic growth factors production.

### Animals

All animal procedures were in accordance with the declaration of Helsinki and with the Guide for the Care and Use of Laboratory Animals and were approved by the Experimental Animal Committee at the Jagiellonian University. Genetically diabetic C57BLKS mice homozygous for a mutation in the leptin receptor (Lepr^db^) were obtained from Jackson Laboratories (Bar Harbor, Maine USA). Animals were 14-week-old at the start of the experiments. Diabetic mice were obese, weighing 45 ± 5 g, hyperglycaemic with glucose concentrations in excess of 400 mg per 100 ml. The hyperglycaemia produced classic signs of diabetes, including polydipsia, polyuria, and glycosuria. Animals were housed individually, maintained under controlled environmental conditions (12-h light/dark cycle at approx. 23°C), and provided with standard laboratory food and water ad libitum.

### Experimental protocol

After general inhalatory anesthesia with halothane, hair on the back was shaved. Two full-thickness excisional circular wounds (4 mm in diameter) were made using biopsy punch on the dorsum of each mice. Animals were randomized to receive either PBS, AAV-LacZ, AAV-FGF4-IRES-GFP, AAV-VEGF-A or AAV-FGF4-IRES-VEGF-A. Five animals were included into each group (n = 5). All AAV vectors and PBS were injected in the wound edges immediately after incision through four (2 per each wound) intradermal injections with a total volume of 100 μl. Animals received 3 × 10^10 vp of an appropriate AAV vector.

### Determination of wound area

Two wounds on the dorsum of each mice were photographed and measured using Image J software by an observer blinded to the experimental protocol at day 0 (directly after wounding), day 1 and then every second day till the end of the observation when the last wounds healed (day 21). Ten wounds per each group were included into the analysis. Wound was considered closed when it was completely covered with epithelium. The wound area measured directly after wounding was used as the reference or original area and all further areas were recorded as the percentage of the original area. Once the experimental schedule was completed (day 21) wounded skin, together with a margin of healthy skin, was excised using 8 mm-diameter biopsy punch. One wound was taken for histological examination (n = 5/group) and the second one for determination of transgene level or activity (n = 5/group).

### Detection of β-galactosidase activity

***In situ***: PBS- and AAV-LacZ-injected skin was briefly washed in cold PBS, fixed in 2% buffered formaldehyde and again washed in PBS. AAV-LacZ-transduced cells growing in culture were fixed in 0.25% buffered formalin and washed in PBS. The samples were immersed overnight in a solution containing 1 mg ml^-1 ^5-bromo-4-chloro-3-indolyl-β-D-galactopyranoside (X-gal), 2 mM MgCl_2_, 5 mM K_3_Fe(Cn)_6_, 5 mM K_4_Fe(Cn)_6 _in PBS at 37°C.

***In tissue lysates***: β-galactosidase activity was determined using β-galactosidase Enzyme Assay in PBS- and AAV-LacZ-injected skin according to vendor's protocol. Activity was normalized to the total protein content and expressed in arbitrary units.

### Determination of FGF4 and VEGF-A protein by ELISA

Skin samples were homogenized in 300 μl of lysis buffer (PBS with 1% Triton and protease inhibitors - 10 mM PMSF, 1 mg ml^-1 ^aprotinin and 1 mg ml^-1 ^leupeptin) using an TissueLyser homogenizer (Qiagen). The homogenate was centrifuged at 21 000 g for 10 min at 4°C. The supernatant was collected and used for protein determination using the Bicinchoninic Acid Protein Assay Kit. Analysis was performed with hFGF4- and hVEGF-A-recognizing ELISA kits. The level of hFGF4 and hVEGF-A in conditioned culture medium of AAV-transduced HeLa cells was determined with the same ELISA reagents. The amount of hFGF4 and hVEGF-A was expressed in pg/mg protein (when determined in tissue lysates) and in pg ml^-1 ^(when determined in conditioned cell culture media).

### Histology

Skin from the healed wound beds surrounded by a margin of normal skin and the underlying muscle layer were harvested and fixed in 10% neutral buffered formalin for at least 24 h at room temperature, dehydrated in graded ethanol, cleared in xylene and embedded in paraffin. Perpendicular sections to the anterior posterior axis of the wounds (3 μm thick) were mounted on glass slides, dewaxed, rehydrated with distilled water and stained with haematoxylin/eosin or with Masson's trichrome, according to routine procedures for light microscopy. The areas proximal to the incision were evaluated in all skin sections in 10 random microscopic fields (1000× magnification) by an observer blinded to the experimental protocol. The following parameters were evaluated and scored as previously described [27,28], modified and internally validated in our laboratory: 1) vascularity, 2) granulation tissue formation and remodeling and 3) dermal matrix deposition and regeneration. We used four-point scale to evaluate vascularity (1 - one or two vessels per site; 2 - three vessels per site; 3 - four vessels per site; 4 - five or more vessels per site) and three-point scale to evaluate granulation tissue formation (1 - thin granulation layer with up to 35 cells per site; 2 - moderate granulation layer with up to 45 cells per site; 3 - thick granulation layer with up to 55 and more cells per site) and dermal matrix deposition and regeneration (1 - little collagen deposition and little regeneration with up to 10 hair follicles within the scar; 2 - moderate collagen deposition and moderate regeneration with up to 20 hair follicles within the scar; 3 - high collagen deposition and complete regeneration with up to 30 and more hair follicles within the scar). The edges of the wound in each of the sections were used as comparisons for scoring.

### Immunohistochemistry

To visualize the smallest blood vessels (<10 μm of the inner diameter), skin sections were deparaffinized and subjected to antigen retrieval using 0.05 M sodium citrate buffer (pH 6.0). Capillary endothelial cells were detected with biotinylated *Bandeiraea simplicifolia *I (BS-I) isolectin B_4 _(dilution 1:100, Vector Laboratories; Janki, Poland). Incorporated isolectin was detected with streptavidin- and fluorochrome-conjugated antibodies (Streptavidin Alexa Fluor 546). Additionally, in order to visualize endothelial cell proliferation tissue sections were exposed to proliferating cell nuclear antigen (PCNA) recognizing antibodies (dilution 1:200) followed by fluorochrome-conjugated secondary antibodies (Alexa Fluor 488). All sections were mounted in DAPI (4',6-diamidino-2-phenylindole)-containing medium (a fluorescent stain that strongly binds to DNA).

### Proliferation assay

Mouse dermal fibroblasts were seeded in 96-well plate at confluence 3 × 10^3 ^cells per well and grown in complete DMEM medium containing low (5.5 mM) glucose (cells from WT and db/db mice) or high (25 mM) glucose (cells from db/db mice) for 24 hours. One hour before stimulation complete medium was removed and cells were overlaid with medium containing 0.5% FBS. Cells were stimulated either with rhVEGF-A (50 ng ml^-1^) or rhFGF4 (50 ng ml^-1^) or with both (50 ng ml^-1 ^each) for 24 hours. BrdU incorporation assay was performed according to vendor's protocol.

### Migration assay

Transwell plates (8 μm pore) (Costar, Corning; Poznan, Poland) were coated with fibronectin (20 μg ml^-1^) mixed with 0.5% gelatin in 1:1 ratio. Cells (1 × 10^4 ^per transwell) resuspended in DMEM medium containing low (5.5 mM) glucose (WT and db/db fibroblasts) or high (25 mM) glucose (db/db fibroblasts) supplemented with 0.5% bovine serum albumin (BSA) were applied on the coated transwell plates (upper compartment of a Boyden chamber). Transwell plates with cells were then placed in wells of a 24-well culture dish filled either with low (5.5 mM) glucose (WT and db/db fibroblasts) or high (25 mM) glucose (db/db fibroblasts) DMEM containing 0.5% BSA supplemented either with rhVEGF-A (50 ng ml^-1^) or rhFGF4 (50 ng ml^-1^) or with both (50 ng ml^-1 ^each) (lower compartment of a Boyden chamber). After 20 hours of culture at 37°C each of the transwell plates was washed with PBS, fixed in 10% formalin and stained with haematoxylin and eosin. The non-migratory cells from the filter surface of the upper compartment were gently removed and only the cells that migrated to the lower side were counted in 4 random microscopic fields (200× magnification).

### Quantitative RT-PCR

Total RNA was isolated from cells with Fenozol Total RNA Isolation Reagent (PAA). Synthesis of cDNA was performed using oligo-dT primers for 1 h at 42°C using MMLV reverse transcriptase, according to vendor's instruction. Quantitative RT-PCR was performed in a Rotor Gene RG-3000 (Corbett Research) in a mixture containing SYBR Green PCR Master Mix (SYBR Green qPCR Kit), 50 ng of cDNA and specific primers in a total volume of 15 μl. The primers recognizing MMP-9 (5'-TGTGGATGTTTTTGATGCTATTG-3' and 5'-CGGAGTCCAGCGTTGCA-3'), Flt-1 (5'-GCACCTATGCSTGCAGAGC-3' and 5'-TCTTTCAATAAACAGCGTGCTG-3') and EF2 (5'-GCGGTCAGCACAATGGCATA and 5'-GACATCACCAAGGGTGTGCAG-3') were used. EF2 (elongation factor 2) was used as a housekeeping gene. After incubation for 15 min at 95°C, a three-step cycling protocol (30 s at 95°C, 45 s at 60°C and 45 s at 72°C) was used for 40 cycles. The melting curve analysis was done using the program supplied by Corbett Research. Relative quantification of gene expression was calculated based on the comparative C_T _(threshold cycle value) method (ΔC_T _= C_T gene of interest _- C_T housekeeping gene_). Comparison of gene expression in different samples was performed basing on the differences in ΔC_T _of individual samples (ΔΔC_T_).

### Statistical analysis

Results are expressed as mean ± SEM unless otherwise stated. One-way analysis of variance (ANOVA) followed by Bonferroni's post-hoc test or unpaired Student's t-test was used to evaluate the statistical significance between investigated groups. p < 0.05 was considered statistically significant.

## Results

### VEGF-A and FGF4 are efficiently produced by AAV-transduced HeLa cells

HeLa cells were exposed to AAV-LacZ, AAV-VEGF-A, AAV-FGF4-IRES-GFP, and AAV-FGF4-IRES-VEGF-A vectors (Figure 1a) each of them administered at 1 × 10^3 ^MOI. This dose of vectors did not influence the cell viability (data not shown). The analysis of gene expression was performed 72 h after transduction. As judged from LacZ staining (Figure 1b) the *in vitro *transduction efficiency with this dose of vectors was not very potent (about 3.5%) but high enough to see the overexpression of all introduced genes (Figure 1b, c, d). Since VEGF-A and FGF4 are secreted proteins [6,21], their expression was measured by ELISA in the culture supernatants collected from transduced and non-transduced HeLa cells (Figure 1c and 1d, respectively). In adults, FGF4 is produced only under pathological conditions by certain cancer cells, while HeLa cell line has been characterized as non-expressing FGF4 [29]. In our hands, control (non-transduced and AAV-LacZ-transduced) HeLa cells also did not release FGF4 into the cell culture media (Figure 1d), while they release about 2616 ± 48 pg ml^-1 ^of human VEGF-A (Figure 1c). Transduction with control vector (AAV-LacZ) did not significantly affect this production which was about 2916 ± 50 pg ml^-1 ^(Figure 1c). When AAV-VEGF-A or AAV-FGF4-IRES-VEGF-A were added to the cells the production of VEGF increased about 2-fold - up to 5667 ± 165 pg ml^-1 ^and 5471 ± 34 pg ml^-1^, respectively (Figure 1c). Interestingly, the localization of hVEGF-A gene after CMV or IRES sequence in the vector did not influence this protein production, as in both cases it was comparable. Unlike hVEGF-A, hFGF4 production was much lower and reached 56 ± 4 pg ml^-1 ^and 254 ± 17 pg ml^-1 ^after transduction with AAV-FGF4-IRES-GFP and AAV-FGF4-IRES-VEGF-A, respectively (Figure 1d). The experiments revealed that both VEGF-A and FGF-4 were released from the cells (data not shown) what confirmed previously published observations [21].

### Wound closure is significantly accelerated after AAV-VEGF-A and AAV-FGF4-IRES-VEGF-A administration

Mice homozygous for a mutation in the leptin receptor (Lepr^db^) exhibit a phenotype similar to adult-onset diabetes mellitus (type II), including a significant wound-healing impairment when compared with their non-diabetic littermates [30,31]. In this study, a 4-mm full-thickness excisional wound model was used. Animals were randomized to receive either PBS, AAV-LacZ, AAV-FGF4-IRES-GFP, AAV-VEGF-A or AAV-FGF4-IRES-VEGF-A (see Figure 1a). Although one of the vectors (AAV-FGF4-IRES-GFP) expressed two proteins - therapeutic (FGF4) and control (GFP) we decided to use additional β-galactosidase (LacZ) expressing vector as the most appropriate control for our study. First of all, LacZ was shown to be less immunogenic than GFP [32]. This seems to be of great importance in wound healing studies as prolonged and dysregulated inflammatory phase results in poor healing [33]. Moreover, IRES-dependent gene expression in bicistronic vectors was shown to be lower than cap-dependent gene expression [25]. Since our therapeutic genes were mostly cap-dependent (except VEGF-A in AAV-FGF4-IRES-VEGF-A vector) we decided to use a control vector carrying LacZ gene under the strong constitutive CMV promoter. Additionally, presence of GFP sequence in AAV-FGF4-IRES-GFP vector served as a control for VEGF-A used in the second AAV-FGF4-IRES-VEGF-A bicistronic vector.

**Figure 2:**
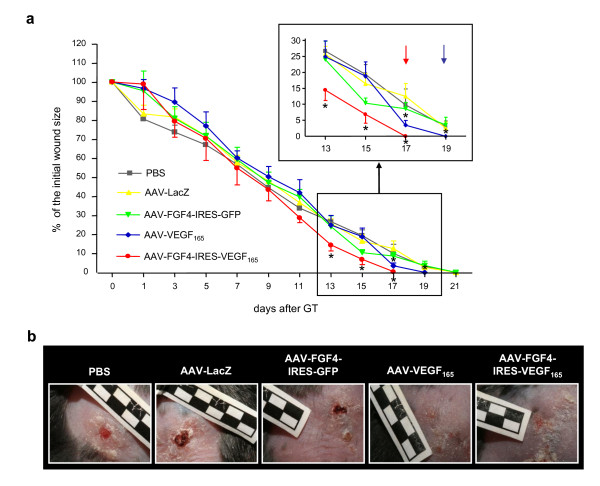
**AAV-VEGF-A and AAV-FGF4-IRES-VEGF-A accelerates time to wound closure in db/db mice**. (A) Quantification of the wound area at consecutive days. Reduction of the wound area after AAV-VEGF-A injection was significantly enhanced starting from day 17. At day 19 all wounds in AAV-VEGF-A group were covered with epithelium and considered closed (arrow, inset). The reduction of the wound area after AAV-FGF4-IRES-VEGF-A injection was even more visible when compared to AAV-LacZ-injected controls starting already from day 13. At day 17 all wounds in AAV-FGF4-IRES-VEGF-A group were covered with epithelium and considered closed (arrow, inset). No acceleration of the wound closure was observed after AAV-FGF4-IRES-GFP at any time-point and the last wounds in this group were considered closed at day 21 together with PBS- and AAV-LacZ-injected animals. (B) Representative pictures taken at day 19 showing wounds of AAV-VEGF-A and AAV-FGF4-IRES-VEGF-A-injected animals completely covered with epithelium and prolonged healing process in PBS-, AAV-LacZ- and AAV-FGF4-IRES-GFP-treated mice. Graph represents means ± SEM (n = 10 wounds/group); *p < 0.05 vs PBS and AAV-LacZ.

The lesions were analyzed at different time-points by measuring the wound area. Neither AAV-LacZ nor AAV-FGF4-IRES-GFP accelerated wound closure at any stage of the healing process (Figure 2a). In late stages of the healing process wounds treated either with AAV-VEGF-A or AAV-FGF4-IRES-VEGF-A healed significantly faster confirming a crucial role of VEGF-A in this phenomenon (Figure 2a). The reduction of the wound area after AAV-VEGF-A injection was clearly visible starting from day 17: 3.48 ± 1.47% of the initial wound area vs 9.87 ± 4.95% in PBS group (p < 0.05) and vs 12.5 ± 4.05% in AAV-LacZ group (p < 0.05). At day 19 all wounds in AAV-VEGF-A group were covered with epithelium and considered closed (Figure 2a, b). Interestingly, the reduction of the wound area after AAV-FGF4-IRES-VEGF-A injection was even more potent starting already from day 13: 14.49 ± 3.29% of the initial wound area vs 26.65 ± 0.16% in PBS group (p < 0.05) and vs 25.7 ± 2.37% in AAV-LacZ group (p < 0.05) at day 13; 6.86 ± 2.75% of the initial wound area vs 19.35 ± 3.07% in PBS group (p < 0.05) and vs 16.42 ± 3.2% in AAV-LacZ group (p < 0.05) at day 15. At day 17 all wounds in AAV-FGF4-IRES-VEGF-A group were covered with epithelium and considered closed. The healing process in some animals from PBS, AAV-LacZ as well as AAV-FGF4-IRES-GFP groups was prolonged until day 21 (Figure 2a).

### Transgene expression in wounds of db/db mice 21 days after AAV transduction

**Figure 3:**
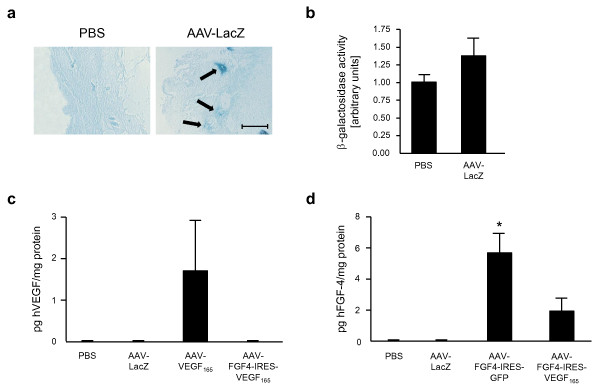
**Weak transgene expression in the skin of db/db mice 21 days after wounding and gene transfer**. (A) Representative skin sections demonstrating β-galactosidase activity (arrows) in AAV-LacZ injected animal and the negative control (PBS treated skin). (B) Colorimetric assay showing slightly increased β-galactosidase activity in skin tissue homogenates from AAV-LacZ-injected animlas in comparison to the PBS-treated mice. (C) hVEGF and (D) hFGF4 protein in skin tissue homogenates measured by ELISA. Graphs represent means ± SEM (n = 5 animals/group); *p < 0.05 vs PBS and AAV-LacZ. Scale bar = 0.05 mm.

To study the location and the time course of AAV expression in wounds β-galactosidase activity was determined by histological analysis and using colorimetric assay in skin lysates of AAV-LacZ injected mice 21 days after treatment. Skin samples from PBS group served as negative controls. Local β-galactosidase activity was observed in histological skin sections close to the sites of wounding and gene transfer. The blue staining was present mostly in the dermal layer and hair follicles (Figure 3a). The colorimetric assay in tissue lysates indicated weak statistically not significant increase in the β-galactosidase activity when compared to the PBS injected animals (Figure 3b).

Expression of both therapeutic genes in skin lysates was determined at day 21 using ELISA kits recognizing hVEGF-A and hFGF4 proteins (Figure 3c, d). Slight increase in the level of hVEGF was detected after AAV-VEGF-A administration (1.7 ± 1.22 pg/mg protein) (Figure 3c). hVEGF protein was not detected in the skin of AAV-FGF4-IRES-VEGF-A-injected mice with available ELISA kit (Figure 3c). In case of hFGF4 its level in skin homogenates of diabetic mice after AAV-FGF4-IRES-GFP injection was a bit higher (5.66 ± 1.27 pg/mg protein) than after AAV-FGF4-IRES-VEGF-A (1.94 ± 0.84 pg mg^-1 ^protein) (Figure 3d). Of note, despite the higher production of hFGF4 from AAV-FGF4-IRES-GFP, the acceleration of wound healing was faster in mice receiving AAV-FGF4-IRES-VEGF-A, indicating for the significance of combined growth factors delivery.

### Local AAV-FGF4-IRES-VEGF-A delivery promotes wound healing at the histological level

**Figure 4:**
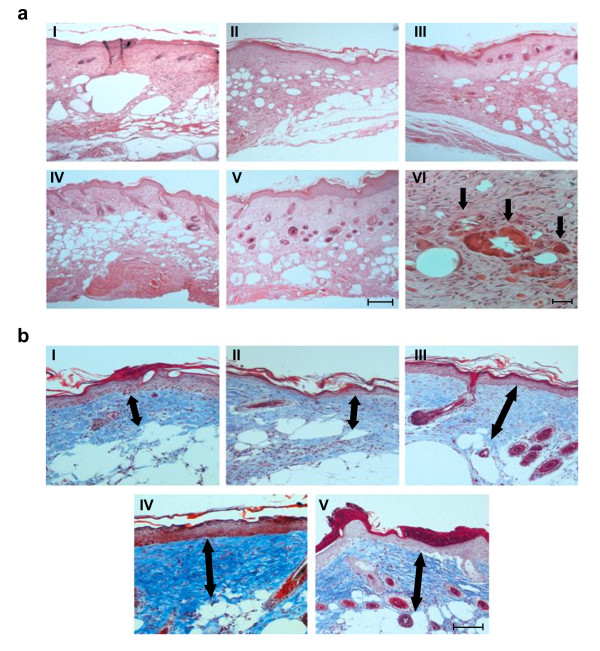
**Skin morphology of db/db mice 21 days after wounding and gene transfer**. (A) Haematoxylin/eosin staining of the skin injected with (I) PBS; (II) AAV-LacZ; (III) AAV-FGF4-IRES-GFP; (IV) AAV-VEGF-A and (V) AAV-FGF4-IRES-VEGF-A. Analysis revealed less adipose tissue and better organized granulation tissue with the presence of hair and restoration of normal architecture of dermis in AAV-VEGF-A and AAV-FGF4-IRES-VEGF-A-treated mice in comparison to PBS-, AAV-LacZ- and AAV-FGF4-IRES-GFP-injected animals. Panels are representative of 5 animals per group. Scale bar (I-V) = 0.1 mm. (VI) Higher magnification of AAV-FGF4-IRES-VEGF-A-injected skin with granulomas (arrows). Scale bar (VI) = 0.05 mm. (B) Representative Masson's trichrome staining of the skin injected with (I) PBS; (II) AAV-LacZ; (III) AAV-FGF4-IRES-GFP; (IV) AAV-VEGF-A and (V) AAV-FGF4-IRES-VEGF-A. Double-headed arrows indicate the thickness of the collagen layer that was significantly thicker after injection of all three therapeutic vectors (AAV-FGF4-IRES-GFP, AAV-VEGF-A, AAV-FGF4-IRES-VEGF-A) when compared to PBS- and AAV-LacZ-treated animals. Panels are representative of 5 animals/group. Scale bar = 0.05 mm.

Diabetic animals usually have a thicker epithelial layer than normal mice [27]. In addition, the different layers are less differentiated and adipose infiltrates are present in the dermis, impairing the normal elasticity of the skin and, as a consequence, it is more prone to a delayed healing [27]. At day 21 all wounds were already covered with epithelium therefore, by histological evaluation, we were not able to observe any differences in the grade of re-epithelialization between analyzed groups of animals. Nevertheless, the epithelial layer covering AAV-VEGF-A- and AAV-FGF4-IRES-VEGF-A-treated wounds was thicker and had a greater cell density when compared to PBS or AAV-LacZ controls or AAV-FGF4-IRES-GFP-treated wounds (Figure 4a, b, photos IV and V).

Interestingly, within the scar tissue of most of the analyzed skin sections we found clusters of inflammatory cells forming granulomas (Figure 4a, photo VI). Granuloma represents a special type of inflammatory reaction in which collection of immune cells is trying to destroy a foreign substance. Apparently, this immune response does not seem to be related to any of the introduced transgenes or AAV capsid proteins as granulomas were found within the healed wounds of all investigated groups of animals including mice injected with PBS. The real cause of such inflammatory reaction is not known and we presume that it might be related to wounding-induced cholesterol crystals formation.

**Figure 5:**
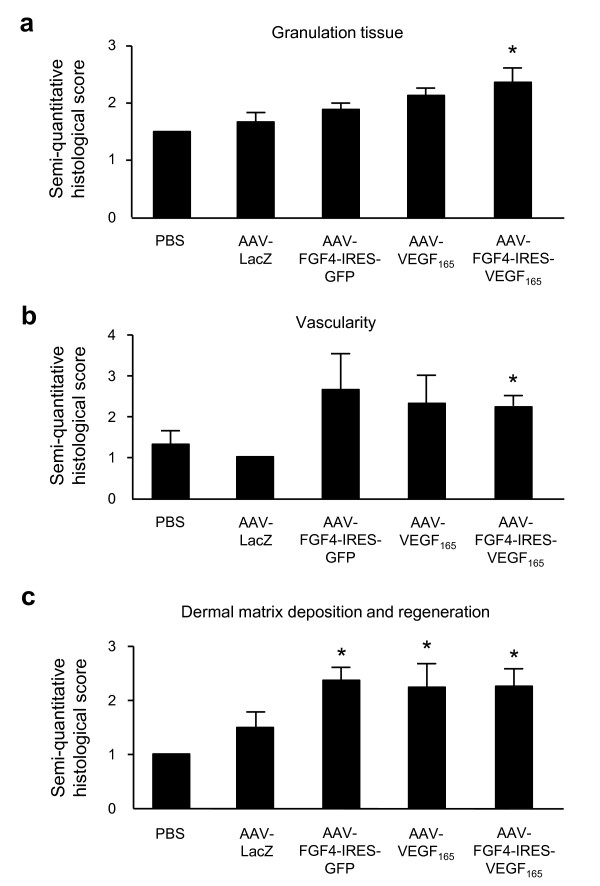
**Semi-quantitative evaluation for granulation tissue**: (A) granulation tissue, (B) vascularity, and (C) dermal matrix deposition and regeneration. Graphs represent means ± SEM (n = 5 animals/group); *p < 0.05 vs PBS and AAV-LacZ.

Although, plenty of inflammatory cells could still be found in the skin sections, most of the cells within the scar tissue of all analyzed groups were of mesenchymal origin (fibroblasts and myofibroblasts). It indicates that the process of tissue remodeling has already been initiated. Granulation tissue and especially its vascularity was enhanced after all three therapeutic vectors in comparison to the control PBS- and AAV-LacZ-injected animals however, statistically significant difference was observed only after bicistronic AAV-FGF4-IRES-VEGF-A vector administration (Figure 5a and 5b, respectively). Thus, VEGF-A delivered in combination with FGF4 into the wound edge reduced adipose substitution and produced a significant improvement in the healing process by increasing the thickness and vascularization of granulation tissue. Additionally, single (AAV-FGF4-IRES-GFP and AAV-VEGF-A) or combined (AAV-FGF4-IRES-VEGF-A) gene transfer resulted in abundant collagen deposition in comparison to the PBS- or AAV-LacZ-treated control wounds (Figure 5c).

### AAV-VEGF-A stimulates new blood vessels formation in the skin of db/db mice

**Figure 6:**
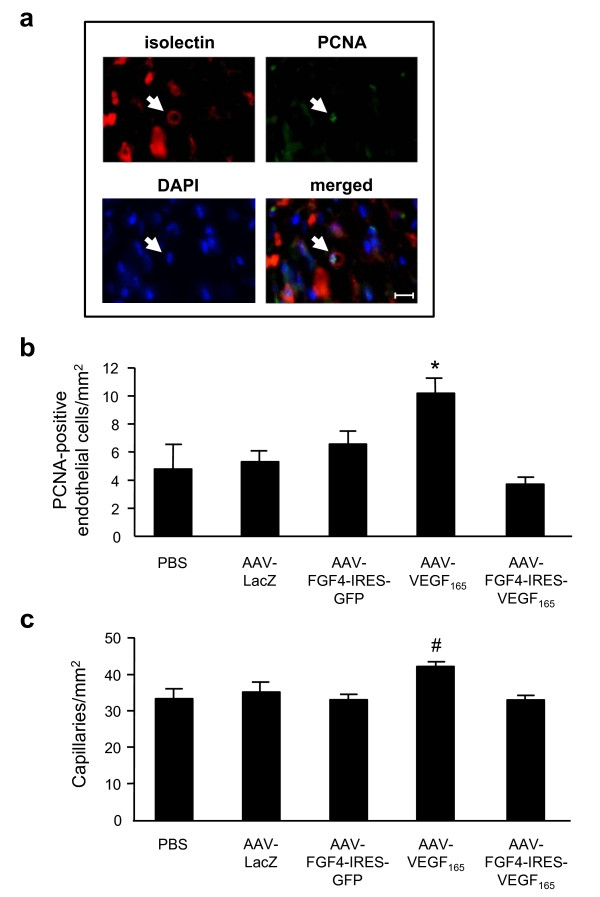
**AAV-VEGF-A stimulates skin neovascularization in db/db mice 21 days after wounding and gene transfer**. (A) Representative pictures demonstrating isolectin B4 binding and expression of proliferating cell nuclear antigen (PCNA) (arrows). DAPI was used to confirm the nuclear localization of PCNA (arrow). (B) The number of proliferating capillary endothelial cells was increased after AAV-VEGF-A injection. (C) Total number of capillaries was also increased only after AAV-VEGF-A administration. Graphs represent means ± SEM (n = 5 animals/group); *p < 0.05 vs PBS and AAV-LacZ; # p < 0.05 vs PBS. Scale bar = 0.01 mm.

Isolectin B_4 _was used to visualize the smallest blood vessels (capillaries) in the skin tissue 21 days after wounding and gene transfer (Figure 6a). Double immunofluorescent staining using biotinylated isolectin B_4 _and PCNA-recognizing antibodies revealed that administration of AAV-VEGF-A stimulated angiogenesis by induction of proliferation of capillary endothelial cells in the dermal area proximal to the healed incision (10.2 ± 1.06/mm^2 ^vs 4.77 ± 1.77/mm^2 ^in PBS group; p < 0.05 and vs 5.31 ± 0.8/mm^2 ^in AAV-LacZ group; p < 0.05) (Figure 6b). This was paralleled with increased total number of capillaries (42.47 ± 1.37/mm^2 ^vs 33.5 ± 2.7/mm^2 ^in PBS group; p < 0.05 and vs 35.41 ± 2.9/mm^2 ^in AAV-LacZ group; p = 0.09) (Figure 6c). The number of skin capillaries detected 21 days after wounding and AAV-FGF4-IRES-GFP or AAV-FGF4-IRES-VEGF-A injection did not differ significantly from PBS- and AAV-LacZ-treated animals (Figure 6c).

### In vitro characteristics of healthy (WT) and diabetic (db/db) mouse dermal fibroblasts

Our observation that AAV-FGF4-IRES-VEGF-A and AAV-VEGF-A accelerated time to wound closure in mice prompted us to explore the underlying mechanisms. As the efficiency of growth factors *in vivo *could result from sustained production by AAV vector which occurred during entire healing process and would require much more animals to check in details, we decided to investigate the migratory and proliferation capabilities of fibroblasts using recombinant growth factors.

**Figure 7:**
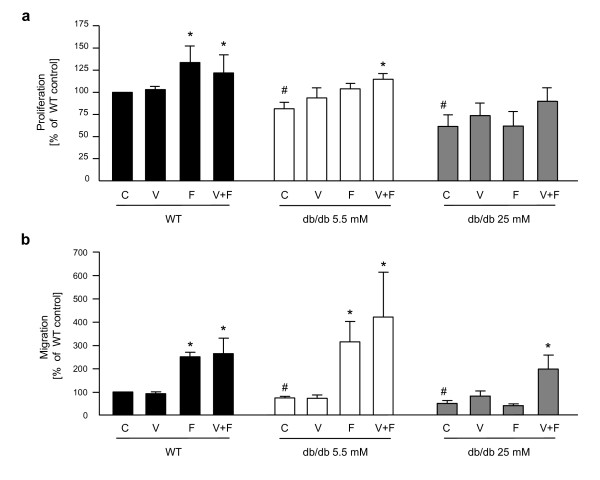
**Combined rhFGF4 and rhVEGF-A treatment improves high glucose-impaired biological properties of diabetic mouse dermal fibroblasts**. Functional *in vitro *tests with age- and passage-matched fibroblasts isolated from the skin of diabetic and wild-type mice: (A) Proliferation after 24 hours of culture. Diabetic fibroblasts cultured in 5.5 mM glucose DMEM (empty bars) show impaired basal proliferation in comparison to wild-type fibroblasts (black bars) which is even more intense when the db/db cells are kept in 25 mM glucose (gray bars). rhFGF4 (delivered alone or in combination with rhVEGF-A) induces WT cell proliferation whereas only co-stimulation with rhVEGF-A significantly increases proliferation of cells isolated from db/db mice and cultured in 5.5 mM glucose (similar tendency in diabetic fibroblasts kept in 25 mM glucose). (B) Migration after 20 hours of culture. Diabetic fibroblasts cultured in 5.5 mM glucose DMEM (empty bars) show impaired basal migration on gelatin/fibronectin in comparison to wild-type fibroblasts (black bars) which is even more intense when the cells are kept in 25 mM glucose (gray bars). Migration towards rhFGF4 gradient (delivered alone or in combination with rhVEGF-A) is preserved in WT cells and in cells from db/db mice cultured in 5.5 mM glucose. Fibroblasts isolated from the skin of db/db mice and cultured in 25 mM glucose show impaired migration towards rhFGF4, that can be restored by combined rhFGF4 and rhVEGF-A treatment. C - control, V - rhVEGF-A (50 ng ml^-1^), F - rhFGF4 (50 ng ml^-1^), V+F - rhVEGF-A (50 ng ml^-1^) and rhFGF4 (50 ng ml^-1^). Graphs represent means ± SEM from n = 5 (A) and n = 3 (B) independent experiments performed in duplicates; *p < 0.05 vs appropriate control; # p < 0.05 vs WT control; § p < 0.05 vs WT and db/db 5.5 mM control.

Proliferation of diabetic and wild-type fibroblasts was measured using BrdU incorporation assay (Figure 7a). Diabetic fibroblasts cultured in low glucose (5.5 mM) DMEM proliferated slightly but significantly slower than wild-type cells (81.5 ± 7.4% vs 100%, respectively; p < 0.05). Fibroblasts from diabetic mice cultured in high glucose (25 mM) DMEM exhibited more potent reduction in proliferation rate (61.5 ± 13% vs 100% in WT control; p < 0.05 and vs 81.5 ± 7.4% in db/db 5.5 mM control; p < 0.05). rhFGF4 delivered alone or in combination with rhVEGF significantly increased the proliferation of wild-type fibroblasts (133.7 ± 19.2% and 121.8 ± 20.6% vs 100% WT control, respectively). Proliferation of db/db fibroblasts cultured in low glucose in the presence of rhFGF4 was slightly weaker than of WT cells and, although the trend was clearly visible, did not reach the statistical significance (103.5 ± 6.3% vs 81.5 ± 7.4% db/db 5.5 mM control, p = 0.09) (Figure 7a). Interestingly, combined rhFGF4 and rhVEGF-A treatment slightly but significantly increased the proliferation rate of db/db fibroblasts cultured in low glucose (115 ± 6.4% vs 81.5 ± 7.4% db/db 5.5 mM control, p < 0.05). Cells from db/db mice cultured in high glucose concentration did respond neither to rhFGF4 or rhVEGF-A and their proliferation did not change significantly when two growth factors, rhFGF4 and rhVEGF-A, were used (Figure 7a).

Differences in basal migration on fibronectin/gelatin were observed between diabetic (db/db) and healthy (WT) fibroblasts (Figure 7b). Migration of diabetic fibroblasts cultured in low glucose DMEM was impaired when compared to wild-type fibroblasts (73.5 ± 7% vs 100%, respectively; p < 0.05). When diabetic fibroblasts were cultured in high glucose DMEM the impairment of migration was much more potent (49 ± 13% vs 100% in WT control; p < 0.05 and vs 73.5 ± 7% in db/db 5.5 mM control; p < 0.05). Migration in response to single rhFGF4 treatment increased more than 2 times in case of wild-type fibroblasts (249.7 ± 19%; p < 0.05 vs WT control) and about 3 times in case of diabetic fibroblasts cultured in low glucose DMEM (313.5 ± 87.4%; p < 0.05 vs db/db 5.5 mM control). When rhFGF4 was added in combination with rhVEGF-A the migration of both cell types (WT and db/db cultured in low glucose) did not differ significantly from the one observed after single rhFGF4 treatment (264 ± 64%; p < 0.05 vs WT control and 419.4 ± 192.7% vs db/db 5.5 mM control, respectively) (Figure 7b). Migration of diabetic fibroblasts cultured in high glucose DMEM did change neither upon single rhFGF4 nor rhVEGF-A treatment. However, it was strongly increased (about 4 times) in response to both growth factors (196.4 ± 60.4%, p < 0.05 vs db/db 25 mM control) (Figure 7b).

### FGF4 stimulates MMP-9 and Flt-1 expression in primary mouse dermal fibroblasts

**Figure 8:**
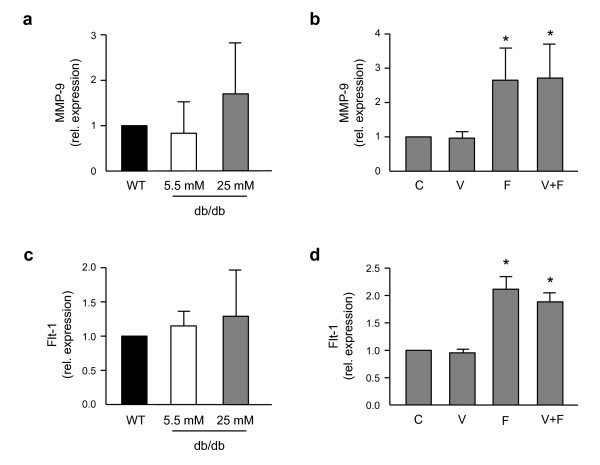
**rhFGF4 stimulates MMP-9 and Flt-1 expression in diabetic mouse dermal fibroblasts**. (A) There are no significant differences in the basal MMP-9 expression between WT and db/db fibroblasts cultured either in low (5.5 mM) or high (25 mM) glucose. (B) MMP-9 expression is up-regulated upon single rhFGF4 or combined with rhVEGF-A treatment in db/db fibroblasts cultured in hyperglycemic conditions. (C) There are no significant differences in the basal Flt-1 expression between WT and db/db fibroblasts cultured either in low (5.5 mM) or high (25 mM) glucose. (D) Flt-1 expression is up-regulated upon single rhFGF4 or combined with rhVEGF-A treatment in db/db fibroblasts cultured in hyperglycemic conditions. C - control, V - rhVEGF-A (50 ng ml^-1^), F - rhFGF4 (50 ng ml^-1^), V+F - rhVEGF-A (50 ng ml^-1^) and rhFGF4 (50 ng ml^-1^). Graphs represent means ± SEM from n = 3 independent experiments performed in duplicates; *p < 0.05 vs control.

Basal MMP-9 expression did not differ significantly between analyzed groups (Figure 8a). Sole rhVEGF-A treatment did not influence MMP-9 expression in diabetic dermal fibroblasts cultured in high glucose concentration (Figure 8b), but it was significantly up-regulated by rhFGF4 (Figure 8b). As there was no additional up-regulation after combined rhFGF4 and rhVEGF-A treatment, this effect most probably depends only on FGF4. Nevertheless, this rhFGF4-mediated stimulation of MMP-9 expression seems to be insufficient to significantly increase the migration of diabetic fibroblasts cultured in 25 mM glucose DMEM, as its rate did not change upon sole rhFGF4 treatment (see Figure 7b). Thus, we have performed analysis of the expression of one of the VEGF receptors, Flt-1, as a possible mechanism responsible for this observation. Similarly to MMP-9, there were no significant differences in the basal Flt-1 expression between WT and db/db fibroblasts kept either in low or high glucose (Figure 8c). Sole rhVEGF treatment did not influence Flt-1 expression in diabetic fibroblasts, but again, as in case of MMP-9, it was up-regulated by rhFGF4 with no further change by combined treatment with rhVEGF-A and rhFGF4 (Figure 8d).

## Discussion

The salient finding of the present study is demonstration that combined overexpression of VEGF-A and FGF4 results in the faster wound healing in diabetic mice than single treatment. This can be a result of enhanced migration of fibroblasts stimulated by two growth factors, the process at least partially dependent on up-regulation of MMP-9 and VEGF receptor 1 expression enhanced by FGF4.

VEGF-A has been shown to play a pivotal role in the initiation of angiogenesis, based on its ability to induce the expression of proteases that digest components of the extracellular matrix that impede angiogenesis, to promote endothelial cell proliferation, and to prevent their apoptosis [6,8]. In addition, it has been demonstrated that VEGF-A gene transfer improves diabetes-impaired wound healing by stimulation of angiogenesis and granulation tissue formation [9,10].

In our study, the healing process after injection of AAV-VEGF-A was also accelerated when compared to PBS- or AAV-LacZ-treated animals. FGF4 delivered alone in a form of AAV-FGF4-IRES-GFP vector did not influence the rate of wound healing. So far, there were no studies investigating the role of this agent in this phenomenon. In contrary to VEGF-A, FGF4 is an oncogene and is not produced in adults under normal, physiological conditions [17]. It is very well known that disruption of the normal process of tissue regeneration in diabetes mellitus is related to the decreased production of different growth factors and VEGF-A is one of them [34-36]. Therefore, FGF4 delivered alone, although possessing the ability to up-regulate the endogenous VEGF-A expression [19,20], may be ineffective in reparative processes in diabetes. In this sense, the combined therapy using FGF4 and VEGF-A coding sequences seemed to be a better approach. In fact, AAV-mediated combined gene transfer of FGF4 and VEGF-A improved diabetic wound healing over the effect exerted by AAV-VEGF-A.

Using available ELISA kits we were not able to detect any up-regulation of hVEGF-A and only slight hFGF4 production in the skin of AAV-FGF4-IRES-VEGF-A-injected mice at the end of the healing process. We suspect that the turnover of transduced cells might result in loss of introduced gene expression over time and finally lead to very low or lack of expression especially that at day 21 all wounds in the AAV-FGF4-IRES-VEGF-A group were healed since couple of days already. Also, as demonstrated by β-galactosidase *in situ *staining and with the colorimetric assay in skin tissue lysates, the expression of the reporter gene 21 days after vector injection was local and rather low. Similar observation has been made by Badillo and co-workers, who used lentiviral vectors, capable of high and stable gene delivery, for the transduction of wounded skin tissue. The authors observed that 14 days post wounding and viral treatment GFP expression was limited to isolated areas or completely absent [37]. Importantly, we did not detect any hVEGF-A and any hFGF-4 in the plasma of the transduced mice (data not shown) what indicates that there was no systemic exposure to these agents and no significant side effects.

In the present study combinatory therapy with VEGF-A and FGF4 coding sequences increased the vascularity of granulation tissue. Concerning vascularity, only mature vessels that contained erythrocytes were counted. In contrast to AAV-VEGF-A combined VEGF-A and FGF4 treatment did not significantly change the number of isolectin-labeled capillaries and their proliferation 21 days after gene transfer suggesting the lack of stimulatory effect on the neovascularization. On the other hand, it is very well known that upon completion of epithelialization (what in case of AAV-FGF4-IRES-VEGF-A-injected animals occurred a couple of days before histological examination) cell proliferation and neovascularization cease, scar tissue forms, and the wound enters the remodeling phase [38]. One of the typical features of transformation of the granulation tissue into scar is regression of vascular structures. Therefore, the lack of effect of AAV-FGF4-IRES-VEGF-A vector on the total number of skin capillaries and their proliferation 21 days after wounding and gene transfer may be explained by the phenomenon of their natural regression.

Very recently, Brem et al. demonstrated that VEGF-A was capable of stimulating the migration of activated keratinocytes over the wound area what indicates that this growth factor can promote epithelialization independently of its role in recruiting and stimulating endothelial cells in the repair process [39]. Although, in the present study stimulation of keratinocyte migration was not investigated, we cannot exclude that it also contributed to the faster wound closure in AAV-VEGF-A- and AAV-FGF4-IRES-VEGF-A-treated mice independently of the stimulation of angiogenesis.

In our hands, VEGF-A delivered alone or in combination with FGF4 into the wound edge produced a significant acceleration of the wound closure that was associated with increased thickness of granulation tissue, increased number of cells within the dermal layer and abundant collagen deposition. Collagen is the major connective-tissue component of granulation tissue, scar and dermis. Its synthesis and deposition are critical for wound closure [39].

Studies demonstrating that diabetes does have effects on essential aspects of fibroblast biology, such as proliferation and collagen synthesis, pointed at the important and complex functions of these cells during tissue repair [33,40,41]. Therefore, in the present study we addressed the question whether glucose concentration might somehow modulate proliferation, migration and angiogenic gene expression in diabetic dermal fibroblasts exposed to sole or combined rhVEGF-A and rhFGF4 treatment. Age- and passage-matched fibroblasts isolated from the skin of non-diabetic (WT) mice of the same background strain served as control. Diabetic fibroblasts were cultured either in low (5.5 mM) or high (25 mM) glucose DMEM concentration and the later was intended to resemble the diabetic environment. We have observed significant differences in basal proliferation and migration on fibronectin/gelatin between diabetic and healthy (WT) mouse dermal fibroblasts. Moreover, db/db fibroblasts cultured in high glucose concentration (25 mM) exhibited even more dramatic reduction in proliferation and migration rate than db/db fibroblasts cultured in low glucose (5.5 mM). Additionally, culture in the presence of high glucose concentration led to significantly impaired response to rhFGF4 treatment. Apparently, co-stimulation with rhFGF4 and rhVEGF-A significantly improved migration of these cells.

We have examined MMP-9 (gelatinase) expression as a possible mechanism responsible for this observation. We did not observe any significant differences in the basal MMP-9 expression between WT and db/db fibroblasts cultured either in low or in high glucose concentration. This is somehow in agreement with other data demonstrating decreased migration of db/db fibroblasts in comparison to WT cells associated with only a selective increase in pro-MMP-9 in db/db fibroblasts but with no difference in active MMP-9 [40].

Interestingly, we found MMP-9 gene to be significantly up-regulated in db/db fibroblasts cultured in high glucose upon rhFGF4 treatment. Similar activity of FGF4 has been demonstrated by Anteby *et al*. in trophoblast suggesting its important role in early placental development [42]. Therefore, we can speculate that by increasing the invasiveness of skin fibroblasts FGF4 might also play a crucial role in the process of wound healing. Sole rhVEGF-A treatment did not influence MMP-9 expression in db/db fibroblasts cultured under hyperglycemic conditions. Moreover, there was no additional up-regulation after combined rhFGF4 and rhVEGF-A treatment, what indicates that this effect depends on FGF4. Nevertheless, FGF4-mediated stimulation of MMP-9 expression seems to be insufficient to significantly increase migration of diabetic fibroblasts kept in high glucose, as its rate did not change upon sole rhFGF4 treatment. As an additional confirmation we have performed the western blot analysis of the levels of pro-MMP-9 in the tissue homogenates. We observed about 3-fold up-regulation of this protein in the skin of animals treated with FGF-4 transgene in comparison to control animals injected with AAV-LacZ. However, due to big variability between the samples the difference was not statistically significant (data not shown).

Wang and Keiser demonstrated that VEGF-A up-regulates MMP-1, -3, and -9 expression in human smooth muscle cells (SMCs) and accelerates their migration through synthetic ECM barriers [43]. Furthermore, the authors showed expression of the high-affinity Flt-1 receptor in human SMCs and its phosphorylation upon VEGF-A treatment, suggesting its role in mediating VEGF-A action. Very recently Brem et al. demonstrated that VEGF-A stimulates migration of human fibroblasts cultured *in vitro*, what indicates a non-angiogenic effect of VEGF-A on wound closure [39]. In our model, we did not observe any significant *in vitro *effect of rhVEGF-A on primary diabetic or non-diabetic mouse dermal fibroblasts migration. Moreover, rhVEGF-A did not change Flt-1 mRNA level in those cells. Interestingly, however, we have found up-regulation of this gene expression in response to rhFGF4 in diabetic fibroblasts cultured in the presence of high glucose concentration. Moreover, there was no difference in Flt-1 expression upon single rhFGF4 or combined with rhVEGF-A treatment, what suggests the FGF4-specific effect.

## Conclusion

On the basis of these findings, we can conclude that VEGF-A may increase migration of diabetic fibroblasts cultured in high glucose concentration through FGF4-mediated up-regulation of one of the VEGF receptors, Flt-1. In this sense, combined therapy approach with VEGF-A and FGF4 genes may significantly improve the delayed wound repair in diabetes over the effect exerted by single VEGF-A treatment. Whether this finding might have important clinical implications remains to be established and deserves further pre-clinical and clinical investigation.

## Abbreviations

AAV: adeno-associated viral vector; db/db, diabetic; Flt-1: fms-like tyrosine kinase-1 receptor (VEGF receptor 1); FGF4: fibroblast growth factor 4; MMP-9: matrix metalloproteinase-9; VEGF-A: vascular endothelial growth factor-A.

## Competing interests

The authors declare that they have no competing interests.

## Authors' contributions

AJ participated in the design of the study, carried out the practical work and drafted the manuscript. PK, JL, AZ, AS, JS, MK, HT, TO, RD and EV participated in the practical work and discussions. SYH and AJ participated in design of the study and helped to draft the manuscript. JD conceived of the study, designed it and edited the manuscript. All authors read and approved the final manuscript.
